# Chronic oral corticosteroids use and persistent eosinophilia in severe asthmatics from the Belgian severe asthma registry

**DOI:** 10.1186/s12931-020-01460-7

**Published:** 2020-08-12

**Authors:** S. Graff, S. Vanwynsberghe, G. Brusselle, S. Hanon, C. Sohy, L. J. Dupont, R. Peche, A. Michils, C. Pilette, G. Joos, R. E. Louis, F. N. Schleich

**Affiliations:** 1 Department of Respiratory Medicine, Ghent University Hospital, Ghent, Belgium; 2 Respiratory Division, Universitair Ziekenhuis Brussel, Vrije Universiteit Brussel (VUB), Brussels, Belgium; 3 Department of Respiratory Medicine, Centre Hospitalier Universitaire UCL Namur, Université Catholique de Louvain, Yvoir, Belgium; 4 Department of Respiratory Medicine, Katholieke Universiteit Leuven, Leuven, Belgium; 5 Department of Respiratory Medicine, CHU-Charleroi, A. Vésale Hospital, Charleroi, Belgium; 6 Chest Department, Erasme University Hospital, Université Libre de Bruxelles, Brussels, Belgium; 7 Cliniques Universitaires St-Luc and Institute of Experimental and Clinical Research, Université Catholique de Louvain, Brussels, Belgium; 8 Department of Respiratory Medicine, CHU Sart-Tilman, I3GIGA Research Group, University of Liege, Liege, Belgium

**Keywords:** Asthma, Blood eosinophils, Oral corticosteroids

## Abstract

**Background:**

Severe asthma (SA) may require frequent courses or chronic use of oral corticosteroids (OCS), inducing many known side effects and complications. Therefore, it is important to identify risk factors of chronic use of OCS in SA, considering the heterogeneity of clinical and inflammatory asthma phenotypes. Another aim of the present analysis is to characterize a subpopulation of severe asthmatics, in whom blood eosinophil counts (BEC) remain elevated despite chronic OCS treatment.

**Methods:**

In a cross-sectional analysis of 982 SA patients enrolled in the Belgian Severe Asthma Registry (BSAR) between March 2009 and February 2019, we investigated the characteristics of the OCS treated patients with special attention to their inflammatory profile.

**Results:**

At enrollment, 211 (21%) SA patients were taking maintenance OCS (median dose: 8 [IQR: 5–10]) mg prednisone equivalent). BEC was high (> 400/mm^3^) in 44% of the OCS treated population. Multivariable logistic regression analysis showed that risk factors for chronic use of OCS in SA were late-onset asthma (i.e. age of onset > 40 yr), frequent exacerbations (i.e. ≥2 exacerbations in the previous year) and non-atopic asthma. Late-onset asthma was also a predictor for persistently high BEC in OCS treated SA patients.

**Conclusion:**

These data showed a significant association between a persistently high BEC and late-onset asthma in OCS treated SA patients. Whether it is poor compliance to treatment or corticosteroid insensitivity the reasons for this association warrants further investigation.

Although severe asthma (SA) represents only 5–10% of the population with asthma [1], the condition accounts for a major part of the financial burden to health care systems posed by asthma [2]. More than half of the incremental cost has been recently attributed to comorbidities [3].

SA defined according to a joint statement from the European Respiratory Society and the American Thoracic Society (ERS/ATS) may require frequent or chronic use of oral corticosteroids (OCS). Indeed, OCS are prescribed as maintenance therapy in 45% of adult SA patients who remain uncontrolled despite GINA step 4–5 treatment [4].

However, OCS are responsible for serious side effects [5] and complications accounting for a significant economic burden [6, 7].

OCS effectively target elements of the T2 inflammatory pathway, suppressing blood eosinophils quite rapidly [8] and decreasing mast cells in airway smooth muscle [9]. Yet some patients still present with high blood eosinophil levels (≥ 400/mm^3^) despite OCS maintenance therapy.

The goals of the present analysis were to compare patients with and without OCS maintenance, and to characterize patients with high levels of blood eosinophils despite this treatment. For this purpose, we used the Belgian Severe Asthma Registry (BSAR), which is a cohort of adult severe asthmatics and aims at enhancing awareness and knowledge on the natural history of SA in order to optimize patient care [10].

## Methods

In a cross-sectional analysis on SA patients enrolled in the BSAR, we compared characteristics of SA patients chronically using OCS with those SA patients who did not. Chronic OCS use was defined as daily use of OCS. Furthermore, we made a comparison of patients with high (≥400/mm^3^) and low blood eosinophil counts (BEC) under OCS.

We also looked at a small number of severe asthmatics with persistently elevated BEC despite OCS therapy in whom an anti-IL5 monoclonal antibody was started.

Between March 2009 and February 2019, severe asthmatics [1] from 21 Belgian centers were enrolled in the BSAR, utilizing a secured web database which admits password protected anonymized data [10], after gaining fully informed written consent (2008/221). The American Thoracic Society definition of SA [11] was used for patients recruited between 2009 and 2014. As the definition of SA changed in 2014 [1], the BSAR website was adapted accordingly.

Asthma was diagnosed based on symptoms of cough, breathlessness or dyspnea together with the demonstration of airflow variability [12]. The latter was defined by one or more of the following: increase in forced expiratory volume in 1 s (FEV1) of 12% or greater after inhalation of 400 μg of salbutamol or an inhaled concentration of methacholine provoking a 20% fall in FEV1 of less than 16 mg/ml. Methacholine challenges were performed according to a standardized methodology as previously described [13].

Other prerequisites for inclusion were: age ≥ 18 years, asthma follow-up by a respiratory physician for at least 12 months, education on the disease provided to the patient, and compliance thought to be satisfactory in order to include only patients with severe refractory asthma and those in whom comorbidities had been addressed. All the data presented were collected at the time point of recruitment into the registry.

Patients were characterized as atopic if they had at least one positive specific IgE (> 0.35 kU/l; ImmunoCAP system, Phadia AB, Uppsala; Sweden) to at least one common aeroallergen (cat, dog, house dust mites, grass pollen, tree pollen and a mixture of molds) or a positive skin prick test. An exacerbation in the previous year was defined by a course of OCS for at least 3 days for asthma worsening. Emergency room visits, hospitalizations, and intensive care unit stays were also recorded. Presence (or absence) of treatment with long-acting muscarinic antagonists (LAMA), short or long-acting ß2-agonists (SABA, LABA), anti-IgE, anti-IL5 and macrolides was recorded, as well as OCS maintenance doses.

Quality of life was assessed using the self-administered Asthma Quality of Life Questionnaire (AQLQ) [14] and asthma control by both the Juniper Asthma Control Questionnaire (ACQ) [15] and the Asthma Control Test (ACT) [16].

Patients underwent FENO measurement at a flow rate of 50 ml/s according to the ERS/ATS recommendations (NIOX, Aerocrine, Sweden) [17] followed by spirometry with bronchodilation (inhalation of 400 μg salbutamol) and complete pulmonary function tests with evaluation of lung volumes and diffusion capacity.

Tobacco status were recorded. An ex-smoker was defined as someone who had stopped smoking for at least 6 months.

Sputum was induced almost exclusively in Liège and processed as previously reported [18]; 225 sputum samples were collected in the data set (23% of the total population). Cell counts were estimated on samples centrifuged (Cytospin) and stained with Hemacolor® Staining set after counting 500 non-squamous cells (Merck chemical, Overijste, Belgium). Sputum cytology was analyzed and 4 phenotypes were defined: the eosinophilic phenotype with ≥3% sputum eosinophil count (and < 76% neutrophil count), the neutrophilic phenotype with ≥76% sputum neutrophil count (and < 3% eosinophil count), and the mixed granulocytic phenotype being a combination of the above [19]. The fourth was paucigranulocytic phenotype, defined as an inflammatory cell count below these thresholds. Blood samples were drawn for evaluation of total serum IgE levels, specific IgE and BEC.

Nasal polyps and sinusitis were diagnosed by Ear Nose and Throat specialists either by endoscopy or sinus CT scanner. Gastroesophageal reflux was diagnosed either upon anamnesis (symptoms of heartburn), or by the presence of oesophagitis upon gastroscopy or treatment response to PPI. Chest CT imaging was obtained in patients with an atypical presentation of SA and in case of history of smoking. Hospital Anxiety and Depression Scale (HADS) questionnaires [20] were used to assess psychopathology.

Data on vocal cord dysfunction, allergic bronchopulmonary aspergillosis, Aspirin-sensitivity, eosinophilic granulomatosis with polyangiitis (EGPA: formerly called Churg-Strauss syndrome), occupational asthma (asthma acquired in the workplace), premenstrual asthma, and obesity were also collected.

Near-fatal asthma is described as an asthma attack that require intensive care unit stay with mechanical ventilation.

BEC were categorized in 4 groups: < 150/mm^3^, between 150 and 299/mm^3^, between 300 and 400/mm^3^, and > 400/mm^3^ to evaluate eosinophil levels in patients treated with OCS and the mean dose of OCS in each group.

Finally, an analysis was conducted to compare patients normalizing their BEC (< 400/mm^3^) under OCS treatment versus those who did not.

### Statistical methods

Continuous variables are presented as mean and SD when normally distributed or as median and interquartile range (IQR) when not normally distributed. Categorical variables were presented as frequencies and percentages. Continuous variables were compared by t-test when normally distributed or by Wilcoxon Mann-Whitney test when non-parametric. ANOVA or Kruskal Wallis analyses were conducted in order to compare more than 2 groups (categories). Fisher exact test was used to compare qualitative variables.

Predicting factors of “chronic OCS use” were assessed by a logistic regression using independent variables such as atopy, gender, age, body mass index, age of asthma onset, smoking status, pack-year, and measurements of ACQ, FENO, post-bronchodilation FEV_1_%, sputum % eosinophils and neutrophils, BEC, presence of exacerbations, and hospitalizations during the last year and comorbidities such as nasal polyps, rhinosinusitis, bronchiectasis, emphysema, obesity, GERD, allergic bronchopulmonary aspergillosis, vocal cord dysfunction, aspirin-sensitivity, EGPA, psychopathy, occupational, premenstrual, or Aspergillar asthma. Chronic OCS use was used as the dependent variable. After examining for all potential predictors, the univariate association with the outcome, a stepwise backward logistic regression was conducted; initial model included variables with an association of *p* < 0.20. Then, the best predictive model was conducted deleting those variables that had the weakest association with the outcome (*p* > 0.05). This analysis (logistic regression) was repeated in order to identify predictors of BEC ≥ 400 mm^3^.

A *p* value < 0.05 was considered statistically significant. Statistical analysis was done using STATA version 14.0 (Statistical Software, College Station, TX: StataCorp LP).

## Results

Between March 2009 and February 2019, 982 severe asthmatics that fulfilled the definition of SA [1, 11] were enrolled in the BSAR.

### Characteristics of OCS maintenance treated SA patients

Demographic, functional and inflammatory characteristics and comorbidities of SA patients are shown in Table 1. At enrollment in the BSAR, 211 (21%) SA patients were taking maintenance OCS (median dose (IQR): 8 (5–10) mg prednisone equivalent/day). The proportion of males was higher (*p* = 0.02) in SA patients treated with maintenance OCS and these patients significantly more often had late-onset (≥40 yr) asthma (*p* < 0.0001), were less atopic (*p* < 0.0001), and presented with higher FENO (*p* = 0.03) and higher exacerbation rates (*p* < 0.0001) compared to OCS naïve SA patients. OCS-treated SA patients also had worse asthma control (*p* = 0.03) and asthma-related quality of life scores (*p* = 0.03). Moreover, comorbidities such as emphysema (*p* = 0.002), bronchiectasis (*p* = 0.001), GERD (*p* = 0.007) and EGPA (*p* < 0.0001) were more prevalent in the chronic OCS-treated SA group.

**Table 1:** Comparison of SA patients with or without chronic OCS treatment (*n* = 982)

Characteristics	No chronic OCS use	Chronic OCS use	*P*-value
N (%)	771 (79)	211 (21)	N/A
Gender: F (%) (*n* = 982)	463 (60)	107 (51)	0.018
Age (*n* = 981)	53 ± 16	55 ± 17	0.0792
Age of onset (%) (*n* = 982)			
< 12 yr	241 (32)	41 (20)	< 0.0001
12–40 yr	296 (39)	71 (34)	
≥ 40 yr	222 (29)	97 (46)	
BMI (*n* = 982)	28 ± 13	27 ± 5	0.2454
Smoking History: (*n* = 982)			
Never (%)	471 (61)	126 (60)	0.092
Current (%)	62 (8)	9 (4)	
Ex (%)	238 (31)	76 (36)	
Estimated number of packyears (*n* = 386)	15 (7–25)	15 (7–25)	0.7406
Atopic status: y (%) (*n* = 982)	554 (72)	119 (56)	< 0.0001
Respiratory Familial History of asthma: true (%) (*n* = 982)	348 (45)	70 (33)	0.001
Current housing: (*n* = 975)			
City (%)	241 (31)	57 (28)	0.201
Countryside (%)	281 (37)	70 (34)	
Sub-urban (%)	246 (32)	80 (39)	
SABA: y (%) (*n* = 982)	656 (85)	176 (83)	0.3080
LABA: y (%) (*n* = 982)	755 (98)	211 (100)	0.4520
LAMA: y (%) (*n* = 982)	20 (3)	8 (4)	0.2380
OCS dose (median IQR) (%)			
< 4	N/A	29 (14)	N/A
4–8		76 (36)	
8–16		72 (34)	
> 16 mg/d		34 (16)	
Anti-IL5: y (%) (*n* = 982)	63 (8)	36 (17)	< 0.0001
Anti-IgE: y (%) (*n* = 982)	140 (18)	31 (15)	0.1410
Macrolides: y (%) (*n* = 982)	4 (1)	1 (1)	0.7070
ACT (*n* = 758)	14.0 ± 5.3	12.9 ± 5.2	0.0195
ACQ (*n* = 642)	2.5 ± 1.3	2.8 ± 1.4	0.0319
AQLQ (*n* = 686)	4.2 ± 1.4	3.9 ± 1.4	0.0259
Exacerbations in last 12 months (n = 966)	2 (0–3)	3 (1–4)	< 0.0001
≥3 OCS burst for asthma exacerbation in last 12 months (*n* = 966)	235 (31)	128 (60)	< 0.0001
Number of emergency visits in last year (*n* = 340)	1 (0–1)	1 (0–2)	0.1177
Number of hospitalizations in last year (*n* = 337)	1 (0–1)	1 (0–1)	0.0690
Near fatal episodes last year (*n* = 99)	0 (0–0)	0 (0–1)	0.2762
Death: (%) (*n* = 982)	5 (0.6)	5 (2.4)	0.043
FEV_1_ (L) (*n* = 953)	2.01 ± 0.81	1.97 ± 0.86	0.5144
FEV_1_ (% predicted) (n = 953)	69 ± 21	67 ± 23	0.1874
FVC (% predicted) (*n* = 953)	87 ± 19	87 ± 23	0.7476
FEV_1_/FVC (% predicted) (n = 953)	64 ± 12	62 ± 13	0.0508
FEV_1_ Reversibility (*n* = 793)	11 ± 14	12 ± 13	0.1299
Total Lung Capacity (*n* = 838)	103 (91–115)	102 (89–113)	0.3674
Functional Residual Capacity (*n* = 692)	115 (96–141)	117 (98–138)	0.9700
Residual Volume (*n* = 782)	137 (110–167)	136 (107–171)	0.4914
DLCO (%) (*n* = 700)	83 ± 21	80 ± 19	0.1038
KCO (%) (*n* = 672)	98 ± 21	97 ± 20	0.6176
PC20M Value (mg/ml) (*n* = 88)	1.1 (0.26–8.00)	0.34 (0.10–2.00)	0.1338
Total serum IgE (kU/l) (*n* = 818)	182 (66–506)	162 (66–366)	0.3237
Blood Eosinophils (/mm^3^) (*n *= 800)	290 (120–526)	339 (110–685)	0.2006
Blood Eosinophils (/mm^3^) (*n* = 800)			
< 150	192 (30)	52 (31)	0.071
150–300	133 (21)	22 (13)	
300–400	81 (13)	21 (12)	
> 400	225 (36)	74 (44)	
Sputum eosinophils (%) (n = 225)	7.2 (1.4–37.0)	6.0 (0.4–21.0)	0.1753
Sputum neutrophils (%) (*n* = 225)	51 (29–73)	63 (35–80)	0.3453
Sputum inflammatory (*n* = 225) Phenotypes:			
Paucigranulo	18%	19%	0.450
Eosino (≥3%)	60%	49%	
Neutro (≥76%)	17%	26%	
Mixed	5%	7%	
Exhaled NO (50 ml/sec) (ppb) (*n* = 689)	24 (13–47)	30 (17–58)	0.0269
Emphysema (*n* = 971)	65 (9)	23 (11)	0.002
Bronchiectasis (*n* = 971)	109 (14)	42 (20)	0.001
Rhinosinusitis (*n* = 971)	396 (52)	118 (56)	0.463
Nasal polyposis (*n* = 971)	193 (26)	66 (31)	0.178
Overweight or obesity (*n* = 971)	393 (52)	100 (48)	0.507
Psychopathology (*n* = 971)	140 (18)	39 (19)	0.630
GERD (*n* = 971)	262 (34)	94 (45)	0.007
Vocal Cord Dysfunctions (*n* = 292)	1 (0.5)	1 (1)	0.371
ABPA (*n* = 971)	31 (4)	11 (5)	0.679
Aspirine-sensitive asthma (*n* = 971)	46 (6)	18 (9)	0.323
EGPA (Churg Strauss) (*n* = 971)	13 (2)	14 (7)	< 0.0001
Occupational asthma (*n* = 971)	24 (3)	7 (3)	0.1950
Premenstrual asthma (*n* = 971)	6 (1)	1 (0.5)	0.936
Aspergillar asthma (*n* = 292)	7 (3)	4 (6)	0.312

Data are presented as mean ± SD or median and IQR. *BMI* Body Mass Index, *SABA* short acting beta-2 agonists, *LABA* long acting beta-2 agonists, *LAMA* long acting muscarinic antagonists, *OCS* oral corticosteroids, *FEV*_*1*_ forced expiratory volume in 1 , *FVC* forced vital capacity, *TLC* total lung capacity, *FRC* functional residual capacity, *RV* residual volume, *DLCO* diffusing capacity of lung for carbon monoxide, *KCO* transfer coefficient of the lung for carbon monoxide, *PC20M* provocative concentration of metacholine causing a 20% fall in *FEV*_*1*_ ppb, parts per billion, *NO* nitric oxide, ppb, parts per billion, *ACT* Asthma Control Test, *ACQ* Asthma Control Questionnaire, *AQLQ* Asthma Quality of Life Questionnaire, *GERD* gastroesophageal reflux disease, *ABPA* allergic bronchopulmonary aspergillosis, *EGPA* Eosinophilic granulomatosis with polyangiitis, *N/A* not applicable

**Fig. 1:**
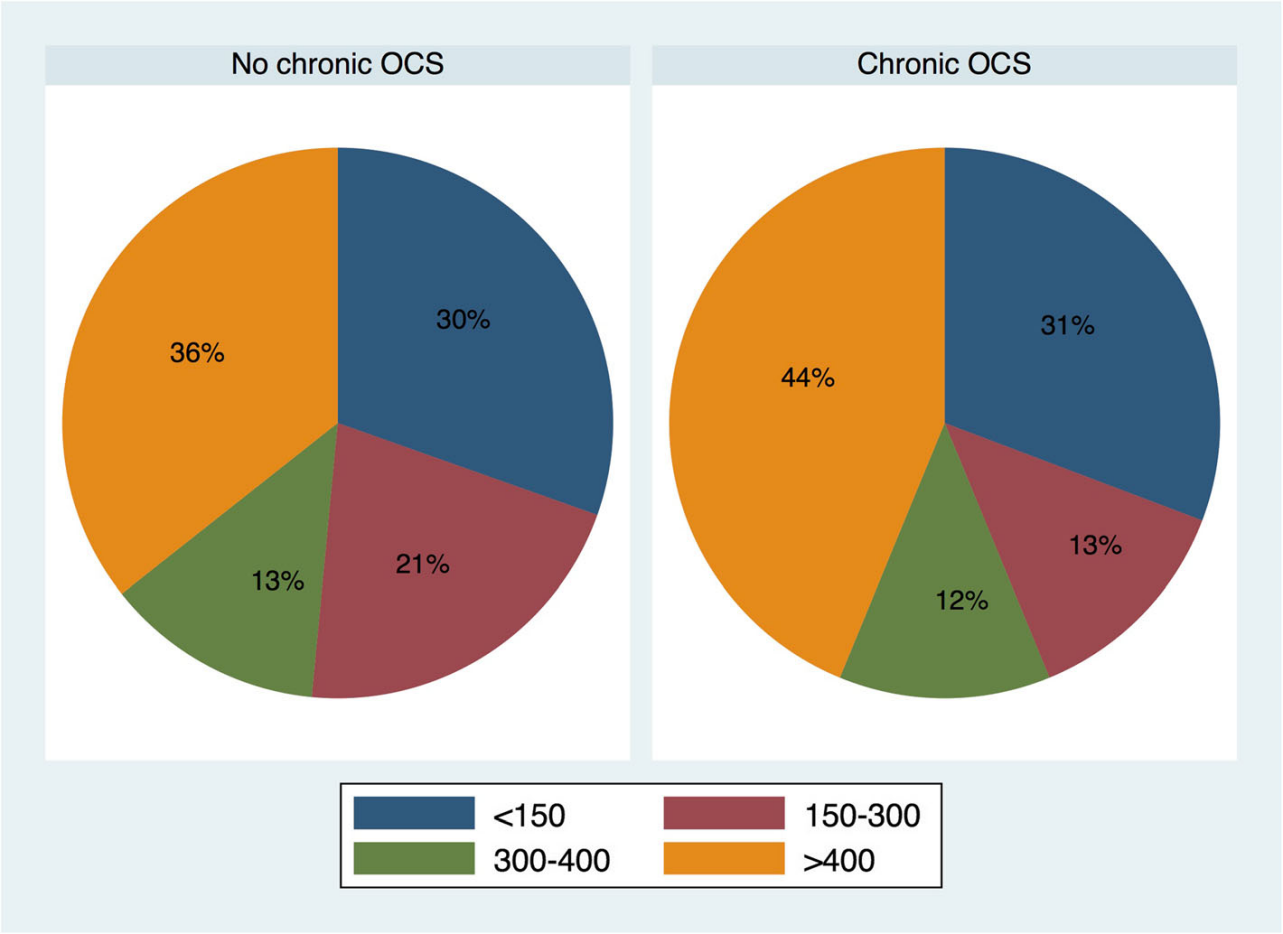
Blood eosinophil count in SA: Proportions of BEC in severe asthmatics according to (presence or absence of) chronic OCS treatment. BEC categories: < 150; ≥150- < 300; ≥300- < 400; ≥400 (/mm^3^)

Interestingly, BEC were similar (*p* = 0.2) between OCS-treated and not treated SA patients with 44% patients with high BEC (> 400/mm3) in the OCS-treated group (Fig. 1). Even in SA patients treated with the highest dose of OCS (> 16 mg/d of prednisone equivalent), 27% still had high BEC.

When selecting only patients on biologics (anti-IL5 or anti-IgE) (*n* = 270) (Table S1), the proportion of males was higher (*p* = 0.001) in SA patients treated with maintenance OCS and these patients were less atopic (*p* = 0.005), and presented with higher exacerbation rates (*p* = 0.0009) compared to OCS naïve SA patients. OCS-treated SA patients also had worse asthma control (*p* = 0.03). Moreover, sputum eosinophils were lower (*p* = 0.02) and sputum neutrophils higher (*p* = 0.005) in the OCS treated patients.

### Predictors of OCS maintenance use

The results of the univariate logistic regression analysis showed an increased risk of using maintenance OCS in non-atopic male and between 44- and 64-years old SA patients with late-onset asthma (i.e. age of onset ≥40 yr), frequent exacerbations (i.e. ≥ 2 exacerbations in last 12 months), GERD and EGPA.

Multivariable logistic regression analysis (Table 2) showed that late-onset asthma (OR [95%CI]:1.98 [1.27–3.08], *p* = 0.003), frequent exacerbations (OR [95%CI]:3.73 [2.41–5.77], *p* < 0.0001) and non-atopic status (0.59 [0.42–0.84], *p* = 0.003) were predictive of chronic use of OCS in SA.

**Table 2:** Factors associated with maintenance OCS use. Results of the logistic regression – Multivariable analysis – Backward stepwise predictive model (*p* < 0.2)

Chronic OCS use	Total population (*n* = 953)					
	UNIVARIATE			MULTIVARIATE		
	OR	95%CI	*P*-value	OR	95%CI	*P*-value
Atopy	0.51	0.37–0.69	< 0.0001	0.59	0.42–0.84	0.0030
Age of onset						
(< 12)			< 0.0001			0.0034
≥ 12; < 40	1.41	0.93–2.15		1.20	0.77–1.86	
≥ 40	2.57	1.71–3.86		1.98	1.27–3.08	
Exacerbations in last 12 months						
(< 0)			< 0.0001			< 0.0001
≥ 0; < 2	1.16	0.73–1.87		1.16	0.72–1.89	
≥ 2	3.73	2.44–5.70		3.73	2.41–5.77	
Female	0.68	0.50–0.93	0.015			NS
Smoking (Never)						
Current	0.54	0.26–1.12	0.1020			NS
Ex	1.19	0.86–1.65				
Age (< 44)						
≥ 44; < 55	1.70	1.09–2.65	0.0385			NS
≥ 55; < 64	1.86	1.18–2.91				
≥ 64	1.41	0.89–2.23				
Number of hospitalizations in last year (< 0)						
≥ 0; < 1	1.10	0.65–1.85	0.0976			NS
≥ 1	2.01	1.04–3.87				
FEV_1_% (< 53)						
≥ 53; < 68	0.70	0.46–1.07	0.1845			NS
≥ 68; < 83	0.64	0.41–0.96				
≥ 83	0.75	0.49–1.16				
Exhaled NO (50 ml/sec) (ppb)	1.0049	1.00053–1.00948	0.028			NS
Blood eosinophils (/mm^3^) (< 150)						
≥ 150; < 300	0.61	0.35–1.05	0.0815			NS
≥ 300; < 400	0.95	0.54–1.69				
≥ 400	1.21	0.81–1.81				
Nasal polyps	1.37	0.98–1.93	0.067			NS
GERD	1.59	1.16–2.17	0.004			NS
ABPA	1.29	0.64–2.62	0.477			NS
Aspirin sensitive	1.47	0.83–2.60	0.183			NS
EGPA (Churg-Strauss)	4.21	1.94–9.11	0.000			NS

*OR* odds ratio, *CI* confidence interval, *FEV*_*1*_ forced expiratory volume in 1 s, *NO* nitric oxide, *ppb* parts per billion, *GERD* gastroesophageal reflux disease, *ABPA* allergic bronchopulmonary aspergillosis, *EGPA* Eosinophilic granulomatosis with polyangiitis, *NS* not significant

### Characteristics of eosinophilic (BEC ≥400/mm^3^) SA patients treated with maintenance OCS

In the population of OCS treated patients, a comparison was made between patients with BEC ≥400 and < 400 mm^3^.

**Fig. 2:**
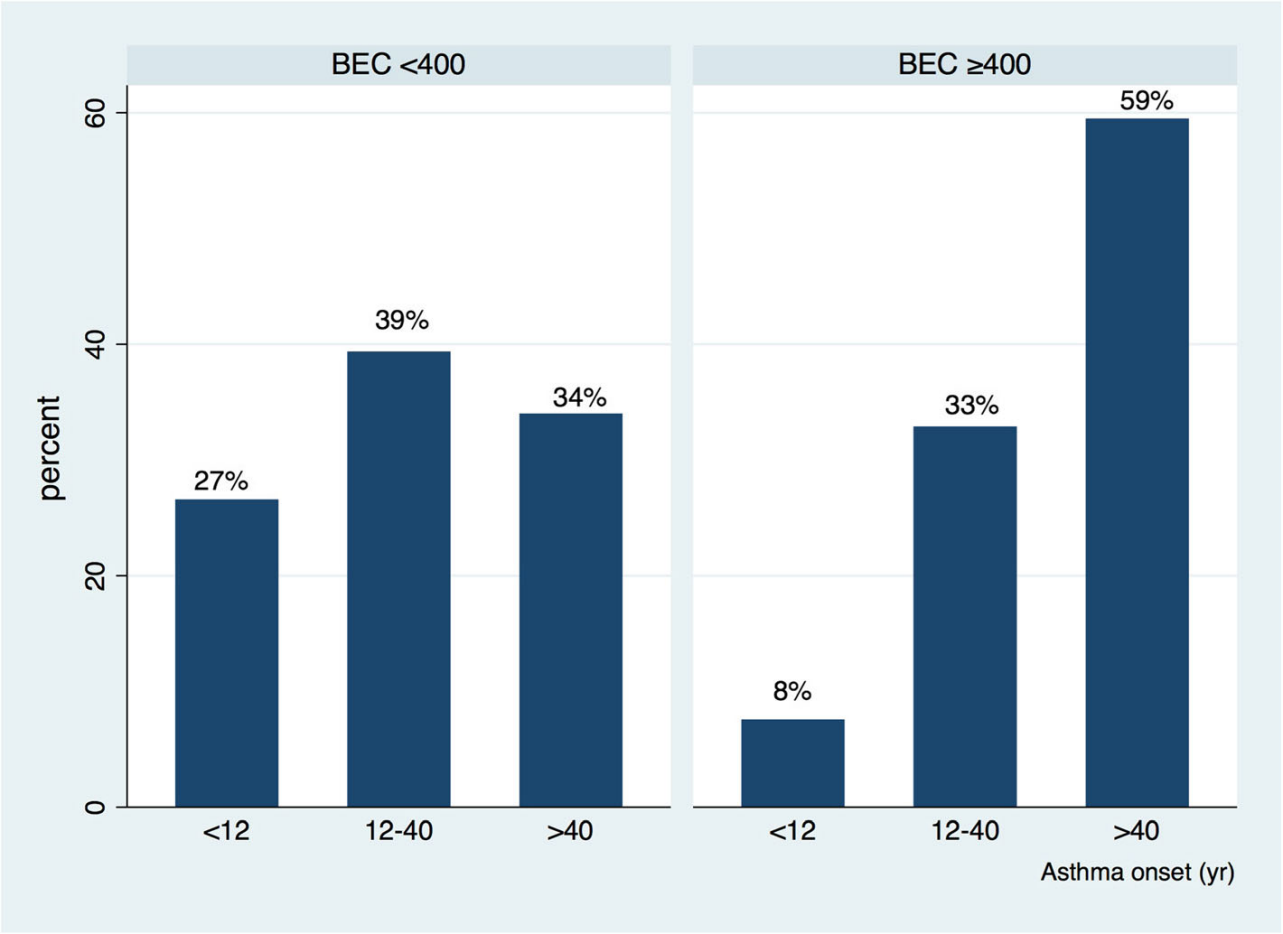
Late-onset asthma predominance and eosinophilia: Distribution of age onset in severe asthmatic with chronic OCS use according to BEC. Age of asthma onset categories: < 12; ≥12- < 40; ≥40 (yr)

Demographic, functional and inflammatory characteristics and comorbidities of the 79 OCS-treated SA patients with high BEC (≥400/mm^3^) were compared to those with low BEC in Table 3. Despite similar OCS doses, eosinophilic patients significantly more often had late-onset asthma (*p* < 0.0001) (Fig. 2), higher levels of FENO (*p* = 0.0005), as well as higher eosinophil (*p* = 0.02) and lower neutrophil (*p* = 0.0004) counts in their sputum than the SA group with a low BEC. These eosinophilic patients also had a higher prevalence of nasal polyposis (*p* = 0.04) and a lower prevalence of anxiety and depression (*p* = 0.004). Moreover, patients with low BEC had slightly poorer lung function (lower FEV_1_ (*p* = 0.04) and FVC% (*p* = 0.009) but similar FEV_1_/FVC and also poorer asthma control (p = 0.04) than the group with a high BEC. These patients also had a higher prevalence of emphysema (*p* = 0.02).

**Table 3:** Comparison of OCS maintenance SA patients with BEC < 400 or BEC ≥ 400 (*n* = 174)

Characteristics	BEC < 400	BEC ≥400	*P*-value
N (%)	95 (55)	79 (45)	N/A
Age of onset (%) (yr) (*n* = 173)			
< 12	25 (27)	6 (8)	0.000
12–40	37 (39)	26 (33)	
> 40	32 (34)	47 (59)	
ACQ (*n* = 61)	2.97 ± 1.29	2.40 ± 1.37	0.0367
FEV_1_ (% predicted) (*n* = 110)	63.7 ± 23.6	71.7 ± 22.4	0.0267
FVC (% predicted) (*n* = 110)	83 ± 21	93 ± 23	0.0088
Sputum eosinophils (%) (*n* = 38)	2.8 (0.4–10.0)	46.5 (4.7–66.8)	0.0223
Sputum neutrophils (%) (*n* = 38)	70.0 (54.0–80.0)	21.7 (9.5–40.5)	0.0004
Exhaled NO (50 ml/sec) (ppb) (n = 67)	23 (13–42)	38 (19–83)	0.0005
Emphysema (based on Chest CT Scanner) (*n* = 174)	15 (16)	7 (9)	0.025
Nasal polyposis (*n* = 174)	25 (26)	33 (42)	0.042
Psychopathology (*n* = 174)	25 (26)	9 (11)	0.004

Data are presented as mean ± SD or median and IQR. *FEV*_*1*_ forced expiratory volume in 1 s, *FVC* forced vital capacity, *ppb* parts per billion, *NO* nitric oxide, *ppb* parts per billion, *ACQ* Asthma Control Questionnaire, *N/A* not applicable

### Predictors of high BEC (≥400/mm^3^) in OCS treated SA patients

The results of the univariate logistic regression analysis showed an increased risk of being eosinophilic despite maintenance OCS in patients with late-onset asthma, high FEV_1_%, high FENO (> 50 ppb), and increased sputum eosinophils (> 21%), but decreased risk are seen in patients with higher sputum neutrophils. Multivariable logistic regression analysis (Table 4) showed that late-onset asthma (OR (IC95%): 31.6 (3.6–276.9), *p* = 0.0047) was a predictor for persistently high BEC in OCS-treated SA patients. However, a high ACQ score (> 3.9) was inversely associated with high BEC (OR (IC95%): 0.17 (0.05–0.63), *p* = 0.0458). Indeed, compared to patients with ACQ scores lower than 3.9 (75th percentile), patients with an ACQ score higher than 3.9 (*n* = 31) were characterized by low BEC, impaired lung function (low FEV_1_) and diffusing capacity of lung for carbon monoxide (DLCO), and a higher rate of emphysema (52%) suggestive of Asthma COPD overlap.

**Table 4:** Factors associated with BEC ≥ 400. Results of the logistic regression – Multivariable analysis - Backward stepwise predictive model (*p* < 0.2)

BEC ≥ 400	Total population (*n* = 174)					
	UNIVARIATE			MULTIVARIATE		
	OR	95%CI	*P*-value	OR	95%CI	*P*-value
Age onset (< 12)						
≥ 12; < 40	2.93	1.05–8.13	0.0009	13.6	1.6–116.8	0.0047
≥ 40	6.12	2.26–16.60		31.6	3.6–276.9	
ACQ (< 1.6)						
≥ 1.6; < 2.9	0.36	0.12–1.11	0.0926	0.29	0.08–1.00	0.0458
≥ 2.9; < 3.9	0.46	0.15–1.39		0.58	0.16–2.08	
≥ 3.9	0.23	0.07–0.76		0.17	0.05–0.63	
Atopy	0.66	0.35–1.20	0.176			NS
Smoking (Never)						
Current	0.53	0.12–2.35	0.0608			NS
Ex	0.46	0.24–0.88				
Age (< 48)						
≥ 48; < 56	1.58	0.66–3.75	0.1448			NS
≥ 56; < 64	0.80	0.34–1.87				
≥ 64	1.97	0.85–4.59				
FEV_1_%	1.02	1.00–1.03	0.029			NS
Exhaled NO (ppb) (< 25)						
≥ 25; < 50	1.83	0.76–4.45	0.0073			NS
≥ 50	4.13	1.70–10.00				
Sputum Eosinophils % (< 0.4)						
≥ 0.4; < 3.0	1.29	0.07–24.39	0.0437			NS
≥ 3.0; < 21.0	0.82	0.04–15.00				
≥ 21.0	15	1.21–185.20				
Sputum Neutrophils %	0.94	0.90–0.98	0.003			NS
Nasal polyposis	1.84	0.96–3.53	0.066			NS

*OR* odds ratio, *CI* confidence interval, *ACQ* Asthma Control Questionnaire, *FEV*_*1*_ forced expiratory volume in 1 s, *NO* nitric oxide, *ppb* parts per billion. *NS* not significant

### BEC before and after anti-IL5 treatment initiation

In a sub-analysis, 12 patients treated with OCS and persistently elevated BEC (≥400/mm^3^) (594 (535–821)/mm^3^), were started on an anti-IL5 monoclonal antibody after enrolment in BSAR. We observed normalization (BEC < 400/mm^3^) of BEC (62 (39–91)/mm^3^) in all these patients after one year of anti-IL5 therapy. Also, as previously shown, the OCS dose was significantly reduced from 8 (7–10) to 4 (2–7) mg daily (*p* = 0.0065) after addition of the biologic therapy.

## Discussion

In this study, we found that approximately one-fifth of SA patients included in BSAR was treated with maintenance OCS and that a high proportion (44%) of these patients still showed a high BEC. The data indicated that late-onset asthma, frequent exacerbations and non-atopic status are associated with OCS maintenance use in SA. Late-onset asthma was also a predictor for persistently high BEC despite OCS.

At enrollment in the registry, 21% of SA patients were taking maintenance OCS, which is a lower proportion of severe asthmatic than previously described by Chung [1]. A partial explanation for this discrepancy could be that in about 26% of this subpopulation, a biotherapy (either IL-5 inhibitors or anti-IgE) had already been started prior to enrolment in BSAR. Indeed, OCS-sparing effects have been reported to be close to 50% with these biotherapies [21–23].

Our data confirms the predominance of females in the overall SA population [10]. However, we found a higher proportion of males in severe asthmatics using maintenance OCS when compared to non-OCS treated patients. A higher proportion of males was previously reported in eosinophilic asthma [24, 25] and eosinophilic asthma has been shown to respond well to chronic inhaled corticosteroids and OCS treatment [26–28].

There are different phenotypes of SA. Among these phenotypes, late-onset non allergic asthma has been found to occur after the age of 40, to be frequently associated with nasal polyps and chronic rhinosinusitis, and to be prone to exacerbations with good response to OCS [25]. In our study, not surprisingly, chronic use of OCS was predicted by late-onset of asthma (≥40 yr), high (≥ 2) exacerbation rates in the previous year, and non-atopic status. Late-onset asthma was also a predictor for high BEC (> 400/mm^3^) in OCS maintenance treated severe asthmatics. Poor response of eosinophils to OCS in this subpopulation needs to be further investigated.

Although OCS are known to suppress eosinophils, BEC were not different between SA patients using maintenance OCS and the ones that do not. We also noticed a high prevalence of patients with high BEC (> 400/mm^3^) in the OCS maintenance treated SA patients. In these patients with high BEC despite OCS maintenance, we may now offer anti-IL5 therapy to decrease the risk of exacerbation [29–31]. Persistently high peripheral blood eosinophils have been previously reported in OCS treated asthmatics [22, 32–34]. MENSA, DREAM and SIRIUS studies show high BEC (MENSA: 320 ± 938 to 280 ± 987); DREAM: 280 ± 1010 to 230 ± 1200; SIRIUS: 250 ± 1245 to 230 ± 1001) despite variable prevalence (25, 30 and 100%, respectively) of patients on OCS maintenance therapy. Whether poor compliance or adherence [35] to treatment or corticosteroid insensitivity are the reasons for high BEC despite OCS maintenance therapy remains unclear.

The lack of measure of compliance/ adherence to treatment represents the first limitation of our study. Poor adherence to treatment is seen in about 32–56% of severe asthmatics [1]. Although compliance was not objectively measured in these severe asthmatic patients, the BSAR investigators only enrolled their patients in the registry if they considered compliance to have been satisfactory in the previous 12 months. We also cannot be absolutely sure that the reported BEC have been measured while patients were actually taking OCS. Indeed, some investigators of BSAR could have provided historical BEC (before starting chronic OCS) or could have stopped OCS in order to obtain a blood eosinophils level > 300/mm3, required for the reimbursement of anti-IL5 in Belgium. Nevertheless, at least the major contributing centers have confirmed that patients were already on OCS maintenance therapy at the time of blood sample collection. Another limitation of our study is the fairly low number of sputums due to the fact that Liège is the only center to use sputum in routine checkup. This could be skewing the data in favor of neutrophilic phenotype for low BEC group, although it is well established that low BEC can have high sputum eosinophils.

In our study, in a small population of patients with eosinophilia despite OCS treatment, we found normalization of BEC under anti-IL5 therapy. With anti-IL5 therapy, a reduction in blood eosinophils is achieved, even in those patients who presented persistent eosinophilia under OCS therapy [36]. More importantly, the introduction of an anti-IL5 treatment allowed an opportunity to reduce the OCS dose in these patients.

Surprisingly, in a fairly small number of patients of this cohort of severe asthmatics, highly uncontrolled asthma was not associated with a high BEC as we could have expected. Instead, it was characterized by impaired lung function (low FEV_1_) and diffusion capacity, and higher rate of emphysema (52%). Emphysema which is associated with lower diffusion capacity, may be a cause of dyspnea, which is a symptom considered in the evaluation of asthma control. These subjects fulfill the profile of patients with Asthma COPD overlap. In contrast, as previously reported [25], some patients with a late-onset eosinophilic phenotype present with low symptom expression despite high inflammation. These are poor perceivers with overestimated asthma control, but are at risk for exacerbations.

## Conclusion

Approximately one-fifth of SA patients were treated with maintenance OCS at enrollment in BSAR. We demonstrated a significant association between late-onset, exacerbations, non-atopic status and chronic use of OCS in this population. Almost half of SA patients treated with chronic OCS have BEC ≥400/mm^3^. Moreover, we show a significant association between high BEC and late-onset asthma in OCS maintenance SA patients.

## Supplementary information

**Additional file 1: Table S1.** Comparison of SA patients on biologics (anti-IL5 or anti-IgE) with or without chronic OCS treatment (*n* = 270).

## Data Availability

The datasets used and/or analyzed during the current study are available from the corresponding author on reasonable request.

## Abbreviations

ACQ: Asthma control questionnaire
ATS: American Thoracic Society
BEC: Blood Eosinophil count
BSAR: Belgian Severe Asthma registry
EGPA: Eosinophilic granulomatosis with polyangiitis, formerly called Churg-Strauss syndrome
ERS: European Respiratory Society
FENO: Fractional exhaled nitric oxide
FEV_1_: Forced expiratory volume in 1 s
FVC: Functional vital capacity
GERD: Gastroesophageal reflux
IL: Interleukin
OCS: Oral corticosteroids
SA: Severe asthma
