# Supplementary material for: Chronic oral corticosteroids use and persistent eosinophilia in severe asthmatics from the Belgian severe asthma registry
Source: Respir Res. 2020 Aug 12;21:214. doi: 10.1186/s12931-020-01460-7 (PMC7424982; doi:10.1186/s12931-020-01460-7)
Supplement: Supplementary file 1 — Additional file 1: Table S1. Comparison of SA patients on biologics (anti-IL5 or anti-IgE) with or without chronic OCS treatment (n = 270). [file 12931_2020_1460_MOESM1_ESM.docx]

**Table S1:** Comparison of SA patients on biologics (anti-IL5 or anti-IgE) with or without chronic OCS treatment (n=270)

| Characteristics | No chronic OCS use | Chronic OCS use | P-value |
| --- | --- | --- | --- |
| N (%) | 203 (75) | 67 (25) | N/A |
| Gender: F (%) | 123 (61) | 25 (37) | 0.001 |
| Age | 53 ± 16 | 53 ± 15 | 0.5938 |
| Age of onset (%)  <12 yr  12-40 yr  ≥40 yr | 67 (33)  73 (36)  61 (30) | 16 (24)  24 (36)  26 (39) | 0.287 |
| BMI | 27 ± 6 | 26 ± 4 | 0.1152 |
| Smoking History:  Never (%)  Current (%)  Ex (%) | 133 (66)  12 (6)  58 (29) | 42 (63)  2 (3)  23 (34) | 0.522 |
| Atopic status: y (%) | 169 (83) | 45 (67) | 0.005 |
| SABA: y (%) | 177 (87) | 56 (84) | 0.289 |
| LABA: y (%) | 203 (100) | 67 (100) | 1 |
| LAMA: y (%) | 10 (5) | 3 (4) | 0.591 |
| OCS dose (median IQR) (%)  <4  4-8  8-16  >16 mg/d | N/A | 13 (19)  22 (33)  22 (33)  10 (15) | N/A |
| ACT | 15.8 ± 5.6 | 13.7 ± 5.7 | 0.0254 |
| ACQ | 2.3 ± 1.8 | 2.5 ± 1.5 | 0.6997 |
| AQLQ | 4.5 ± 1.4 | 4.2 ± 1.6 | 0.3713 |
| Exacerbations in last 12 months | 1 (0 - 3) | 2 (1 - 4) | 0.0009 |
| Number of hospitalizations in last year | 1 (0 - 1) | 1 (0 - 1) | 0.3124 |
| Near fatal episodes last year | 0 (0 - 0) | 0 (0 - 1) | 0.8865 |
| Death: (%) | 3 (1) | 1 (1) | 0.683 |
| FEV_1_ (L) | 2.01 ± 0.81 | 2.16 ± 0.85 | 0.2142 |
| FEV_1_ (% predicted) | 70 ± 19 | 66 ± 21 | 0.1880 |
| FVC (% predicted) | 89 ± 18 | 89 ± 21 | 0.8730 |
| FEV_1_/FVC (% predicted) | 63 ± 12 | 61 ± 13 | 0.1647 |
| FEV_1_ Reversibility | 11 ± 15 | 13 ± 13 | 0.0732 |
| DLCO (%) | 85 ± 21 | 81 ± 20 | 0.3709 |
| PC20M Value (mg/ml) | 0.21 (0.16 – 0.93) | 0.07 (0.04 - 2.00) | 0.4054 |
| Total serum IgE (kU/l) | 298 (136-591) | 300 (156-585) | 0.9608 |
| Blood Eosinophils (/mm^3^) | 269 (100-570) | 340 (126-650) | 0.2997 |
| Blood Eosinophils (/mm^3^)  <150  150-300  300-400  >400 | 52 (33)  31 (19)  13 (8)  60 (38) | 16 (31)  5 (9)  8 (15)  23 (44) | 0.189 |
| Sputum eosinophils (%) (n=28) | 7 (1 - 27) | 0 (1 -3) | 0.0191 |
| Sputum neutrophils (%) (n=28) | 32 (17 – 66) | 72 (60 – 81) | 0.0054 |
| Exhaled NO (50 ml/sec) (ppb) | 26 (13 - 51) | 30 (17 - 79) | 0.1249 |
| Emphysema | 11 (5) | 3 (5) | 0.212 |
| Bronchiectasis | 28 (14) | 12 (18) | 0.223 |
| Rhinosinusitis | 107 (53) | 33 (50) | 0.617 |
| Nasal polyposis | 50 (26) | 17 (26) | 0.702 |
| Overweight or obesity | 102 (50) | 30 (45) | 0.705 |
| Psychopathology | 39 (19) | 9 (13) | 0.667 |
| GERD | 67 (33) | 25 (38) | 0.618 |
| ABPA | 13 (6) | 5 (8) | 0.286 |
| EGPA (Churg Strauss) | 11 (5) | 4 (6) | 0.563 |
| Occupational asthma | 6 (3) | 3 (5) | 0.877 |
